# Increased Serum Beta-Secretase 1 Activity is an Early Marker of Alzheimer’s Disease

**DOI:** 10.3233/JAD-215542

**Published:** 2022-05-03

**Authors:** Roland Nicsanu, Carlo Cervellati, Luisa Benussi, Rosanna Squitti, Roberta Zanardini, Valentina Rosta, Alessandro Trentini, Clarissa Ferrari, Claudia Saraceno, Antonio Longobardi, Sonia Bellini, Giuliano Binetti, Orazio Zanetti, Giovanni Zuliani, Roberta Ghidoni

**Affiliations:** a Molecular Markers Laboratory, IRCCS Istituto Centro San Giovanni di Dio Fatebenefratelli, Brescia, Italy; b Department of Translational Medicine and for Romagna, University of Ferrara, Ferrara, Italy; c Department of Chemical, Pharmaceutical and Agricultural Sciences, University of Ferrara, Ferrara, Italy; d Service of Statistics, IRCCS Istituto Centro San Giovanni di Dio Fatebenefratelli, Brescia, Italy; e MAC-Memory Clinic and Molecular Markers Laboratory, IRCCS Istituto Centro San Giovanni di Dio Fatebenefratelli, Brescia, Italy; f Alzheimer’s Research Unit and MAC Memory Clinic, IRCCS Istituto Centro San Giovanni di Dio Fatebenefratelli, Brescia, Italy

**Keywords:** Alzheimer’s disease, amyloid-β peptide, BACE1, mild cognitive impairment

## Abstract

**Background:**

Beta-site APP cleaving enzyme 1 (BACE1) is the rate-limiting enzyme in amyloid-β (Aβ) plaques formation. BACE1 activity is increased in brains of patients with Alzheimer’s disease (AD) and mild cognitive impairment (MCI) and plasma levels of BACE1 appears to reflect those in the brains.

**Objective:**

In this work, we investigated the role of serum BACE1 activity as biomarker for AD, estimating the diagnostic accuracy of the assay and assessing the correlation of BACE1 activity with levels of Aβ_1 - 40_, Aβ_1 - 42_, and Aβ_40/42_ ratio in serum, known biomarkers of brain amyloidosis.

**Methods:**

Serum BACE1 activity and levels of Aβ_1 - 40_, Aβ_1 - 42_, were assessed in 31 AD, 28 MCI, diagnosed as AD at follow-up (MCI-AD), and 30 controls. The BACE1 analysis was performed with a luciferase assay, where interpolation of relative fluorescence units with a standard curve of concentration reveals BACE1 activity. Serum levels of Aβ_1 - 40_, Aβ_1 - 42_ were measured with the ultrasensitive Single Molecule Array technology.

**Results:**

BACE1 was increased (higher than 60%) in AD and MCI-AD: a cut-off of 11.04 kU/L discriminated patients with high sensitivity (98.31%) and specificity (100%). Diagnostic accuracy was higher for BACE1 than Aβ_40/42_ ratio. High BACE1 levels were associated with worse cognitive performance and earlier disease onset, which was anticipated by 8 years in patients with BACE1 values above the median value (> 16.67 kU/L).

**Conclusion:**

Our results provide new evidence supporting serum/plasma BACE1 activity as an early biomarker of AD.

## INTRODUCTION

Alzheimer’s disease (AD), one of the most common forms of dementia, is a neurodegenerative dis-ease characterized by the loss of function and death of neuronal cells in different brain areas. The progressive formation of extracellular aggregates consisting of the amyloid-β (Aβ) peptide is a pathological hallmark of AD [1, 2]. Aβ peptides assemblies derive from the proteolysis of a transmembrane glycoprotein named amyloid-β protein precursor (AβPP) involving β- and γ-secretase activities [3].

Beta-site APP cleaving enzyme 1 (BACE1) is a β-secretase and it is the rate-limiting enzyme in amyloidogenesis, resulting in Aβ plaques formation. It is mainly expressed by central nervous system (CNS) neurons, but it is found to a lesser extent in peripheral organs [4]. BACE1 appears to have a central function in the pathogenesis of AD: while BACE1 knockout mice show that Aβ production is abolished [5], enzyme over-expression results in Aβ excess—shaped in β-sheet conformation—that forms non-degradable aggregates, facilitating chronic inflammation and neuronal death [6]. Furthermore, BACE1 is also responsible for the generation of N-terminal truncated forms of Aβ, equally toxic forms of the Aβ peptide, whose deposition in the brain appears to occur in the early stages of the disease [7–9].

BACE1 activity is increased in brains of patients with AD and mild cognitive impairment (MCI) and plasma levels of BACE1 appear to reflect those in the brains [10]. It is also expressed and potentially secreted into general circulation by other tissues than the brain: increasing evidences show plasma/serum BACE1 activity increase in AD patients and in MCI converting to AD [11–13].

Recently non-invasive biomarkers development programs reached important achievements employing plasma Aβ_40 - 42_ measured with ultrasensitive technologies [14, 15].

While the association of plasma activity of BACE1 with cerebrospinal fluid (CSF) predictors for progression or conversion to dementia, namely tau protein and Aβ_1–42_ peptide [16, 17] has been previously assessed [11] the association of serum activity of BACE1 with serum levels of Aβ_1 - 40_ and Aβ_1 - 42_ has never been evaluate so far. Our pilot study addresses this topic: first we further investigated the role of BACE1 activity as an early biomarker for AD and estimated the diagnostic accuracy of the assay; then we compared the accuracy of the BACE1 assay with that obtained with serum Aβ forms.

## MATERIALS AND METHODS

### Subjects

In this retrospective study we selected 59 patients with AD (*n* = 31) or with MCI, who were successively diagnosed as AD (MCI-AD, *n* = 28); patients were enrolled at the Memory Clinic (MAC) and at the Operative Unit Alzheimer of the IRCCS Fatebenefratelli, Brescia, Italy according to international guidelines [18, 19] (Table 1). All patients were positive for the core CSF AD biomarkers (Tau, pTau, and Aβ_1 - 42_). Cognitively healthy controls (*n* = 30) were included in the study as control (CTRL) group for comparisons (Table 1). All subjects (or legal guardian) signed informed consent. The study was approved by the Local Ethics Committee (approval number 91/2019, 74/2020).

**Table 1:** Demographic, clinical, and biological presentation of all subjects

	CTRL (*n* = 30)	AD (*n* = 31)	MCI-AD (*n* = 28)	*p*
Sex (% F)	70%	70%	71%	0.963^a^
Age, y (Mean±SD)	71.23±4.81	69.16±10.65	69.89±9.07	0.867^b^
Disease onset, y (Median; 25%; 75%)	–	68.00; 60.00; 75.00	69.50; 65.00; 73.25	0.682^c^
Education, y (Mean±SD)	10.50±4.04	7.06±3.23	7.33±3.63	< 0.001^b^
MMSE (Mean±SD)	27.97±0.32	18.37±5.58	25.15±2.16	< 0.001^b^
Aβ_1 - 42_ CSF (pg/ml) (Mean±SD)	–	353.60±149.60	374.00±122.80	0.572^d^
P-Tau 181 CSF (pg/ml) (Median; 25%; 75%)	–	78.04; 67.65; 114.60	87.41; 56.47; 110.40	0.855^c^
Tau CSF (pg/ml) (Mean±SD)	–	591.50±227.10	626.10±270.50	0.667^d^

Normality assumption was evaluated by Shapiro-Wilk test. CTRL, controls; AD, Alzheimer’s disease patients; MCI-AD, Mild Cognitive Impairment converting to AD; MMSE, Mini-Mental State Examination. ^a^Chi-squared test, ^b^Kruskal-Wallis test, ^c^Mann-Whitney Test, ^d^*t*-test. Median, 25% Percentile, 75% Percentile are reported for the non-normal distributed variables. SD, standard deviation. Disease onset for MCI-AD refers at first diagnosis, before conversion.

### Serum collection

Peripheral blood samples were collected by venipuncture into Vacutainer^®^ tubes without anticoagulant after an overnight fasting. After 30 min of incubation at room temperature, blood samples were centrifuged at 4,650×g for 20 min; serum were then collected in single-use aliquots and stored at –80°C until analysis.

### BACE1 activity assay

BACE1 activity assay was performed by the Department of Biomedical and Specialist Surgical Sciences from University of Ferrara, as described in their work [12]. Briefly, a stock solution of substrate (392μM dissolved in Dimethyl sulfoxide, DMSO, Sigma-Aldrich, Cat. No. D8418) was diluted to 30μM in 50 mM acetate buffer, pH 4.5, 0.1 M NaCl; 100μL were dispensed in triplicate to the wells of a black, flat-bottom microplate (Nunc Cat. No. 237108). After pre-incubation for 10 min at 37°C, the reaction was started by the addition of 5μL of undiluted serum and the fluorescence was read every 30 s for 20 min using excitation and emission wavelengths of 430 nm and 520 nm, respectively, in a Tecan Infinite M200 (Tecan Group, Switzerland) microplate reader. The reaction rates were converted from relative fluorescence units (RFU) per minute to enzyme units (U) by interpolation with a standard curve constructed using known concentrations of the wild-type enzyme (beta-secretase human, Sigma-Aldrich, Cat. No. S4195). Intra-assay and inter-assay percentage coefficient of variation (% CV) were 6.5% (min-max: 2.6–10.9%) and 11.4% (min-max: 9.9–13%), respectively. Intermediate precision was 10.5% (min-max: 6.3–13.5%). The limit of detection was 861 (U/L). The technician who performed the assay was blinded to the group to which the sample belonged, and the samples were randomly assayed on plates to avoid batch effects.

### Serum levels of Aβ_1 - 40_ and Aβ_1 - 42_

Serum levels of Aβ_1 - 40_ and Aβ_1 - 42_ were measured simultaneously using the commercially available Simoa Human Neurology 3-Plex A assay kit (Quanterix, Lexington, MA, USA) on the automated Simoa SR-X analyzer (Quanterix), following the manufacturer’s instructions. Samples were thawed at room temperature for 60 min and centrifuged at 10,000×g for 5 min prior to analyses, as suggested in the user protocol, to prevent any sample debris from interfering in measurement, then diluted 1:4; all samples were analyzed in duplicate.

### Statistical analyses

The size of the sample was calculated by a power analysis on BACE1 activity (kU/L) assumed as the primary outcome and considering as a reference the measurements performed on 151 controls and 115 AD patients. Considering the mean and the standard deviation (SD) values of BACE1 activity of 15.90±5.8 (kU/L) for the control group and 25.08±14.20 (kU/L) for AD patients (using two-tailed Mann-Whitney non-parametric test for differences between two groups, with alpha level = 0.05 and a power of 0.8) we calculated that 24 subjects per group was the minimum size of the sample necessary for this variation to be significant.

Shapiro-Wilk test was performed in all continuous variables for testing normality distribution. Mann-Whitney and *t*-test were used for comparing of the two groups, for non-normally and normally distributed variables respectively. Chi-squared test was used for categorical variables. Kruskal-Wallis with Dunn’s *post-hoc* test and one-way ANOVA with Tukey’s *post-hoc* test was used for multi-groups comparisons. Correlations between variables were analyzed by Pearson’s r or Spearman’s rho coefficients based on their distribution. Moreover, a receiver operating characteristic (ROC) curve was used to examine the diagnostic performance of BACE1 and Aβ_40/42_ ratio in our groups. Comparison of the AUC was assessed by DeLong test. Disease free curves (Kaplan Meier statistics) were used to compare the age at disease onset by levels of serum BACE1 activity. All analyses were performed by SPSS v.26 and significance two tailed *p*-value set at 0.05.

## RESULTS

### BACE1 assay

**Fig. 1:**
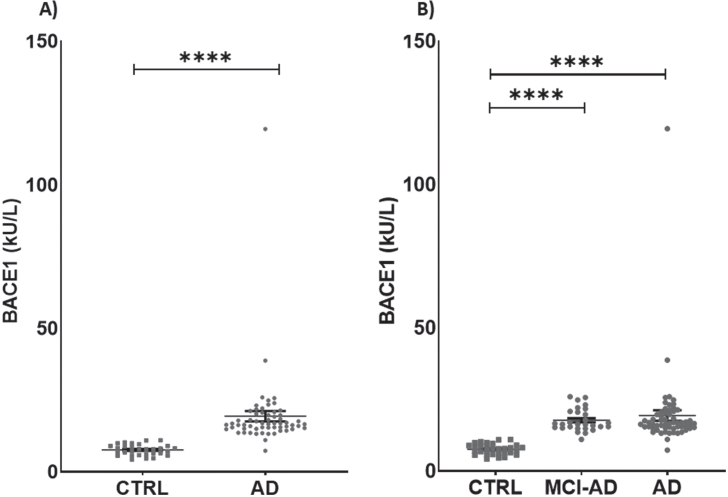
Levels of BACE1 in patients and in controls. A) BACE1 in AD + MCI-AD group (mean±SEM:19.390±1.825) is higher than in CTRL group (mean±SEM: 7.626±0.343, *p* < 0.0001; Mann-Whitney test). B) BACE1 is significantly higher in AD (mean±SEM: 20.88±3.929) and MCI-AD (mean±SEM: 17.73±0.7426) groups as compared to CTRL group (mean±SEM: 7.626±0.343, *p* < 0.0001; Kruskal-Wallis test with Dunn’s multiple comparisons *post-hoc* test). Outliers’ removal (in AD + MCI-AD group in A, and in AD group in B) did not affect the statistical evidence of group differences.

Serum activity of BACE1 was different between groups, being 60.67% higher in AD + MCI-AD than in CTRL [mean±SD: CTRL, 7.626±1.877; AD +MCI-AD, 19.390±14.020; *p* < 0.001] (Table 2; Fig. 1A). The difference was significant also in AD and MCI-AD single groups compared with CTRL (mean±SD: CTRL, 7.626±1.877; MCI-AD, 17.730±3.929; AD, 20.880±19.000; *p* <0.0001) (Table 2; Fig. 1B). Of note, BACE1 serum activity was higher than plasma citrate activity (Supplementary Figure 1A), although the measurements made in both matrices were highly correlated (Supplementary Figure 2A). Thus, we chose to measure the activity of BACE1 in serum, the most suitable sample matrix for assessing enzymes activity, due to the lack of interference from additives.

**Table 2:** Serum biological variables

	CTRL (*n* = 30)	AD (*n* = 31)	MCI-AD (*n* = 28)	*p*
BACE1 (kU/L) (Median; 25%; 75%)	7.85; 5.94; 8.98	16.94; 14.95; 19.72	16.35; 15.09; 20.76	< 0.0001^a^
Aβ_1 - 40_ (pg/ml) (Median; 25%; 75%)	129.40; 90.96; 155.90	110.30; 56.77; 138.80	119.30;83.67; 163.60	0.2612^a^
Aβ_1 - 42_ (pg/ml) (mean±SD)	10.91±0.89	8.56±0.59	8.35±0.77	0.0332^b^
Aβ_1 - 40/42_ (Median; 25%; 75%)	11.42; 9.343; 13.03	12.52; 10.37; 14.74	14.07; 12.22; 16.57	0.0149^a^

Normality assumption was evaluated by Shapiro-Wilk test. ^a^Kruskal-Wallis test, ^b^one-way ANOVA test. Median, 25% Percentile, 75% Percentile are reported for the non-Gaussian distributed variables. SD, standard deviation.

### Aβ_1 - 40_, Aβ_1 - 42_

**Fig. 2:**
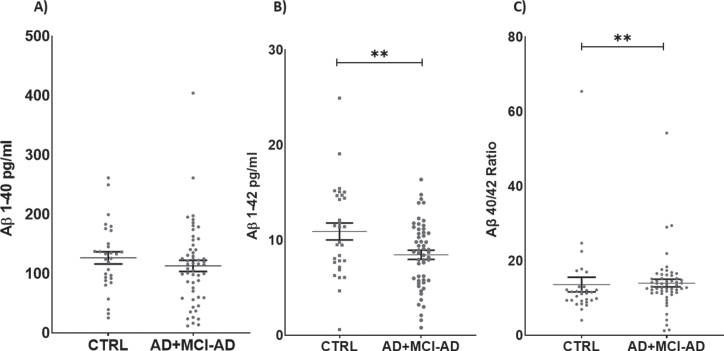
Serum levels of Aβ_1 - 40_ (A), Aβ_1 - 42_ (B), and ratio Aβ_1 - 40/42_ (C). Aβ_1 - 42_ is significantly lower in AD + MCI-AD group (mean±SEM: 8.760±0.476) as compared to CTRL group (mean±SEM: 10.590±0.887, *p* = 0.0090; *t*-test); on the contrary the Aβ_40/42_ ratio is higher in AD + MCI-AD (mean±SEM: 14.01±1.028) as compared to CTRL (mean±SEM: 13.62±1.953, *p* = 0.0042; Mann-Whitney test).

Serum levels of Aβ_1 - 42_ were decreased in AD +MCI-AD patients with respect to CTRL (mean pg/ml±SD: 8.459±3.499 versus 10.910±4.858; *p* =0.0090). Furthermore, we calculated the Aβ_40/42_ ratio, and the analysis revealed a significant in-crease in the AD + MCI-AD group with respect to CTRL (mean±SD: 14.010±7.553 versus 13.620±10.700; *p* = 0.0042) (Fig. 2).

### Correlations

**Fig. 3:**
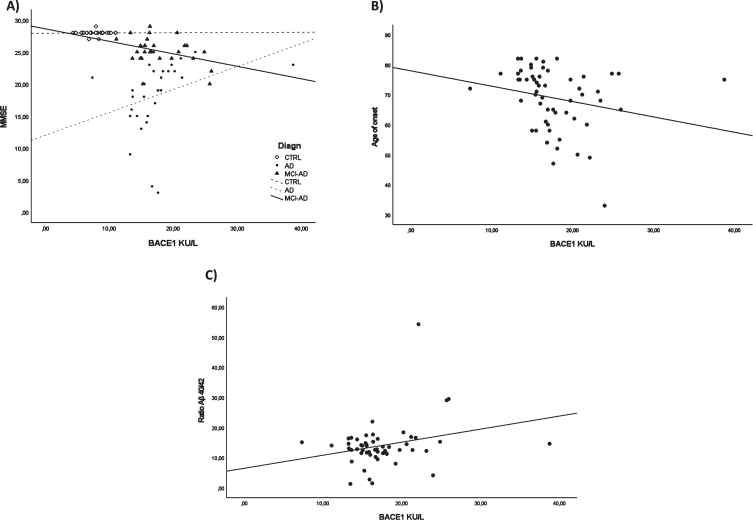
Correlation analysis. BACE1 and MMSE in all the subgroups (A), BACE1 and age of onset in AD + MCI-AD group (B), BACE1 and ratio Aβ_40/42_ in AD + MCI-AD group (C).

To evaluate the association between BACE1 and clinical and biochemical data, we carried out a correlation analysis. MMSE-BACE1 correlation analysis in each study group (CTRL/ AD + MCI-AD /AD) revealed a significant negative correlation only in the AD + MCI-AD group (*r* = –0.424, *p* = 0.035, adjusted for age and sex, Fig. 3A). In AD + MCI-AD, BACE1 levels negatively correlated with the age of onset (Spearman *r* = –0.300; *p* = 0.026, Fig. 3B). Lastly, we found a positive correlation between the Aβ_40/42_ ratio and BACE1 serum levels (Spearman *r* = 0.306; *p* = 0.026, Fig. 3C).

### ROC curve analysis and Kaplan-Meier survival curve

**Fig. 4:**
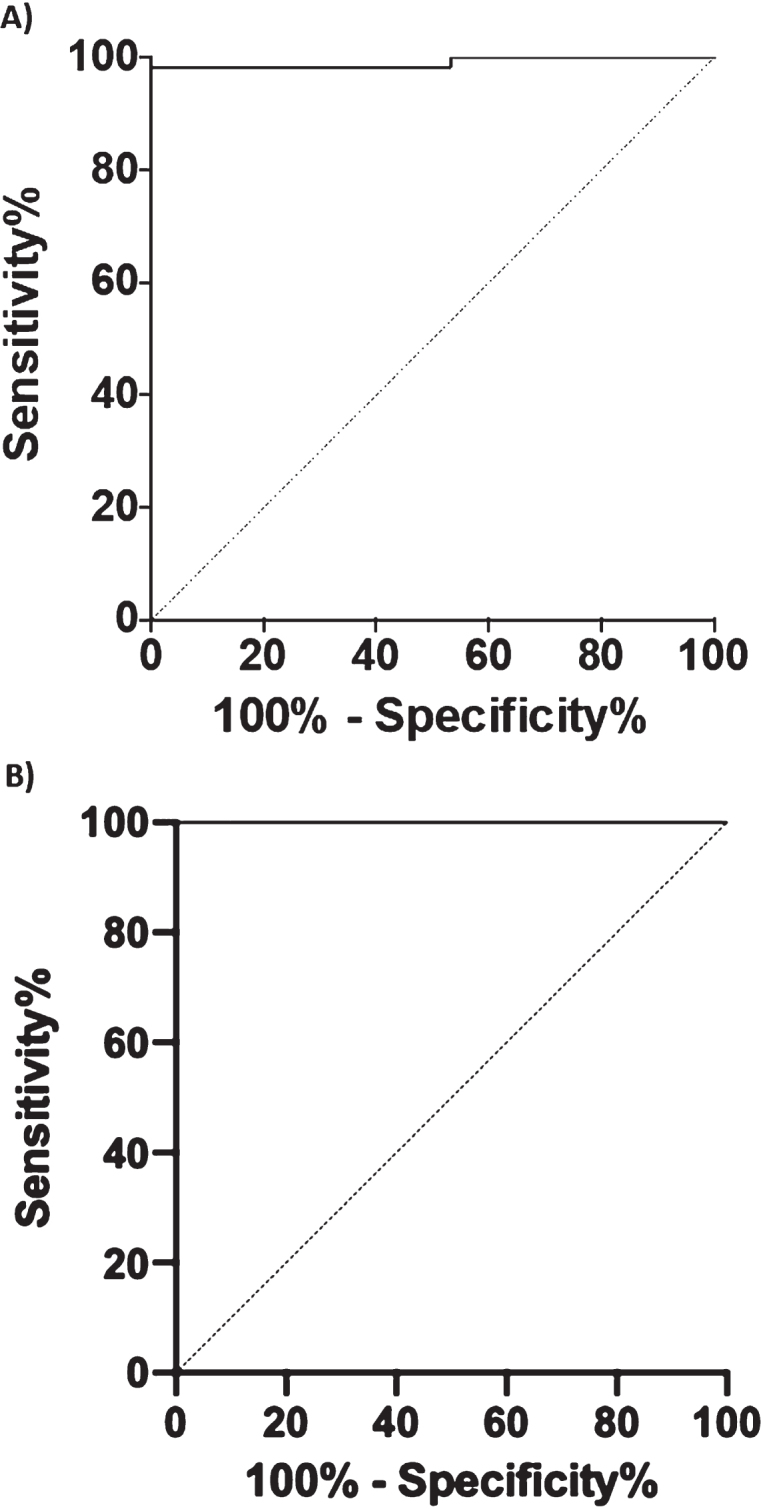
Graphics of the ROC curve analysis. BACE1 levels in human serum in CTRL versus AD + MCI-AD group (A) and CTRL versus MCI-AD (B).

To investigate the diagnostic performance of BACE1 to discriminate CTRL from AD+MCI-AD, we performed a ROC curve analysis (Fig. 4A). The analysis showed an area under the curve (AUC) of 0.991 (CI95%  = 0.9729 to 1.000) with a sensitivity of 98.31% and a specificity of 100% for a cut-off value of 11.04 kU/L. Considering only the MCI-AD group an AUC of 1.000 (CI95%  = 1.000 to 1.000), with 100% of sensitivity and specificity was obtained for the same cut-off value. Moreover, we compared the diagnostic performance of BACE1, Aβ_1 - 42_, and Aβ_40/42_ ratio (DeLong test, ROC_BACE1 versus ROC_Aβ_1 - 42_ *p* < 0.0001 and ROC_BACE1 versus ROC_RATIO Aβ_42/40_: *p*-value = 4.379e-07) attesting a better diagnostic performance of BACE1 assay.

**Fig. 5:**
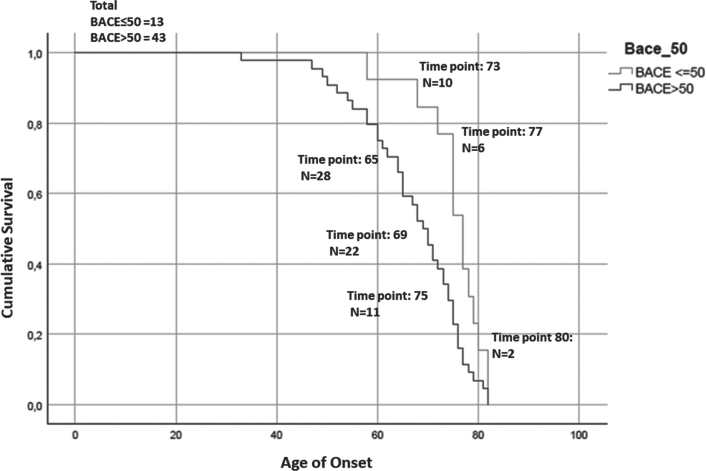
Kaplan-Meier survival curve. For the analysis has been considered the age of onset for AD patients and the age of conversion for the MCI-AD. The number of subjects at risk respectively at: the 25% percentile, median, and 75% percentile of Median time for each group are reported. The test showed that patients with higher serum levels of BACE1 (of the median (16.67 kU/L): BACE > 50) anticipate the pathology of 8 years (77 > 69, *p* = 0.008, Breslow test, BACE < = 50 group: Median time = 77; 25% Percentile = 73.56; 75% Percentile = 80.44; BACE > 50 group: Median time = 69; 25% Percentile = 65.28; 75% Percentile = 72.71).

Finally, in order to evaluate the influence of BACE1 in serum on disease onset and progression, we generated Kaplan-Meier survival curves depicting age at onset (in AD patients) or age at clinical conversion (in MCI-AD) stratified on the basis of their BACE1 activity in serum. We observed a significant anticipation of disease onset in subjects with a serum BACE1 level higher than the median value (77 > 69, *p* = 0.008, Breslow test).

## DISCUSSION

The main result of the current study is that serum levels of BACE1 are increased (higher than 60%) in AD and MCI-AD in comparison with healthy controls and that a cut-off of 11.04 kU/L can discriminate patients with a high sensitivity (98.31%) and specificity (100%). The results further improved when only the MCI-AD individuals were analyzed, showing a sensitivity and a specificity of 100%. Moreover, in the MCI stage BACE1 correlated with disease progression as measured by cognitive decline. These results point out to the high capacity of the BACE1 serum assay to discriminate individuals with a high chance to develop AD at the early stages of the pathology.

Although a small sample size of patient was analyzed, our preliminary results are very encouraging, since they confirm previous evidence of increased levels of BACE1 in serum/plasma [11, 12, 20] and extend existing knowledge showing the correlation with serum Aβ. AD is a multifactorial complex disorder, in which the progressive formation of extracellular Aβ aggregates is a pathological hallmark that occurs a decade or more before symptom onset [21]. Increasing evidences show the potential of serum/plasma BACE1 levels as an early biomarker of cognitive decline [11, 12]. The increase of almost 61% of BACE1 serum activity we achieved, is in line with 53% increase in MCI and 68% in AD showed by Shen and co-authors [11], and of 30% in late onset AD reported by Zuliani et al. (2021) [13].

The cut-off we propose better discriminates AD and MCI individuals from healthy controls than those proposed previously (20.7 KU/L; sensitivity 70% and specificity 75%) [12]. However, our results derive from a sensibly smaller patient sample and consequently have to be taken with caution. Another finding of the current investigation is the association of serum BACE1 levels with age of disease onset. In the same line, we observed a significant anticipation of disease onset (8 years) in subjects with a serum BACE1 level higher than the median value. These findings strengthen the potential of BACE1 assay in serum as an early biomarker for AD. Even though these results should be validated in larger cohorts, they show the potential of this test as a sensitive biomarker that could be employed as an inclusion criterion in phase II clinical trials. The application of this inclusion criterion may help overcoming the limits experienced by BACE1 inhibitor clinical trials, that showed no beneficial effects [22, 23], by identifying people at high risk of conversion in the early stages of the disease. In agreement with the hypothesis that higher levels of serum BACE1 are related to a worsening of the cognitive functions, we found a positive association of cognitive decline with increased levels of serum BACE1. This correlation was also found in two recent reports [11, 12]. Another result is the decrease in serum Aβ_1 - 42_ and increased serum Aβ_40/42_ ratio in AD + AD-MCI group. Moreover, BACE1 activity was positively correlated with serum Aβ_40/42_ ratio in serum. Our results are in line with those showing Aβ_40/42_ ratio as a potential biomarker of prediction of brain amyloidosis [24]. Notably, the discrimination capacity in terms of detecting AD/MCI people is higher for BACE1 with respect to Aβ_40/42_ ratio, suggesting that BACE1 can intercept cognitive worsening at an earlier stage with respect to brain amyloidosis as revealed by Aβ_40/42_ ratio. To this regard, some hints for explanation can be provided by the function that has been proposed for BACE1 as a stress response protein that is upregulated in AD by a number of facilitating factors encompassing oxidative stress, chronic hypoxia [23, 25] or even copper exposure [26]. As a matter of the fact, the transmembrane region of BACE1 holds a highly conserved copper binding domain and overexpression of BACE1 leads to reduced superoxide dismutase activity due to loss of intracellular protein-bound copper [27]. Despite the limitation of a small sample size of the study, current results, achieved in a new AD/MCI population, are consistent with existing literature. Current results provide new evidence supporting serum/plasma BACE1 activity as an early biomarker of AD. We evaluated that 1) serum BACE1 activity increases in AD, 2) it increases at an earlier stage as in MCI-AD, 3) it can detect cognitive decline before AD diagnosis with high sensitivity and specificity, and 4) it performs better than the serum Aβ assay. In addition to the advantages indicated for a good AD biomarker that are, briefly: i) being minimally invasive, ii) being widely accessible, iii) being cost-effective, and iv) being able to reproduce a relevant pathophysiological process of the disease, our study suggests that the measurement of serum BACE1 activity could be employed with beneficial effects for patient classification. In particular, this biomarker could be employed to identify potential responders in upcoming disease-modifying phase II clinical trials (mainly BACE inhibitors or other targeted anti-amyloid therapies), identifying eligible participants at an earlier stage of cognitive decline, before the processes of amyloidosis begin.

## Supplementary Material

Supplementary Material

## DATA STATEMENT

The datasets of raw data generated for this study can be found in the Mendeley Data Repository (doi: 10.17632/dkypt3rpcy.1).
