# Supplementary material for: Increased Serum Beta-Secretase 1 Activity is an Early Marker of Alzheimer’s Disease
Source: J Alzheimers Dis. 2022 May 3;87(1):433–41. doi: 10.3233/JAD-215542 (PMC9198762; doi:10.3233/JAD-215542)
Supplement: Supplementary Material [file jad-87-jad215542-s001.pdf]

Supplementary Material

Increased Serum Beta-Secretase 1 Activity Is an Early Marker of Alzheimer's Disease

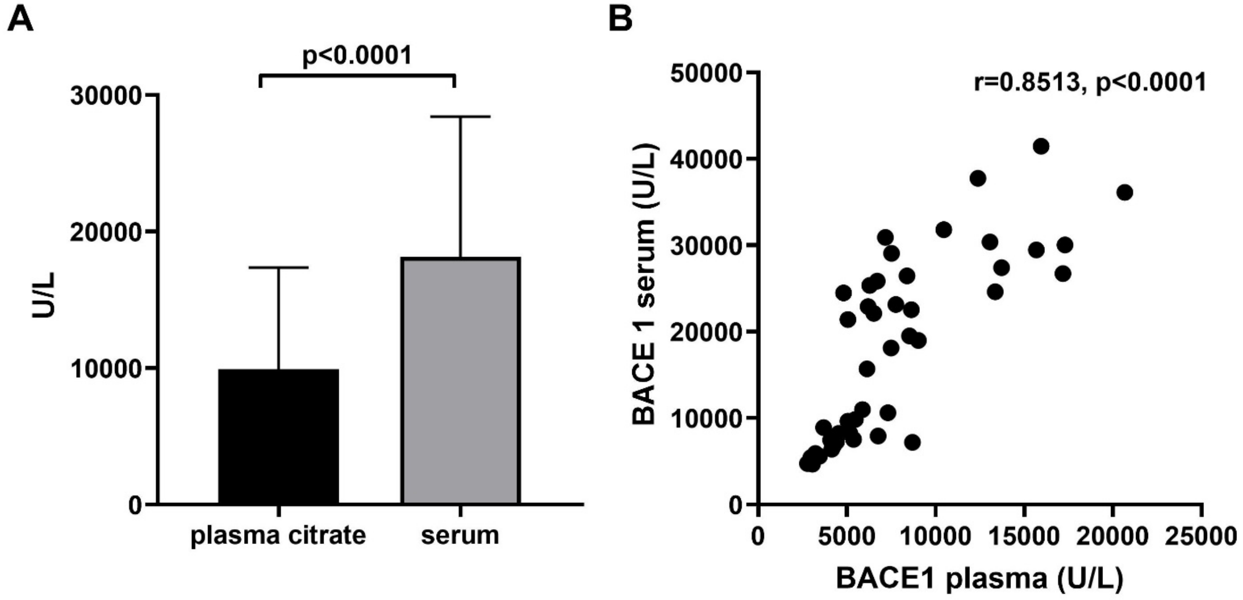

Supplementary Figure 1. BACE1 activity measured in plasma citrate and serum (A) and correlation between the two measurements performed in 55 paired samples from healthy subjects. As displayed, BACE1 activity in serum is higher than in plasma citrate ($p<0.0001$), but the two measurements were highly correlated ($r=0.8513$, $p<0.0001$).
